# Management of Newly Diagnosed Chronic Myeloid Leukemia During COVID-19 Hospitalization: A Teaching Case

**DOI:** 10.7759/cureus.24093

**Published:** 2022-04-13

**Authors:** Ayrton I Bangolo, Jeffin Cherian, Parul Jandir, Quratulain Nasir, Abraham Lo

**Affiliations:** 1 Internal Medicine, Palisades Medical Center, North Bergen, USA; 2 Internal Medicine, Hackensack Meridian Health Palisades Medical Center, North Bergen, USA

**Keywords:** leukemoid reaction, case report, vaccine, covid-19, cml

## Abstract

Chronic myeloid leukemia (CML) is a myeloproliferative neoplasm characterized by the dysregulated production and uncontrolled proliferation of mature and maturing granulocytes. CML has the potential to cause secondary immunodeficiency in affected patients. COVID-19 infection has been associated with worse outcomes in immunocompromised patients, including patients with hematologic cancers, requiring hospitalization. Herein we present a 61-year-old male with known COVID-19 infection who presented for the evaluation of acute hypoxic respiratory failure and was found to have marked leukocytosis of 125,000. The patient was eventually diagnosed with CML, and his respiratory failure resolved with conventional COVID-19 pneumonia treatment. With this case report, we hope to assist clinicians in the workup of marked leukocytosis in the setting of COVID-19 pneumonia and aim to help clinicians in the management of patients admitted with COVID-19 pneumonia and concomitant CML.

## Introduction

Chronic myeloid leukemia (CML) is a clonal disorder of hematopoiesis that arises in a hematopoietic stem or early progenitor cell [1]. The clinical hallmark of CML is the uncontrolled production of mature and maturing granulocytes [2]. About 30% to 50% of patients with CML are asymptomatic at diagnosis, with routine blood tests revealing marked leukocytosis [3]. A recent study by Belsky et al. revealed worse outcomes in immunocompromised patients, including hematologic cancer patients, hospitalized with COVID-19 infection [4]. Here, we present a 61-year-old male admitted for acute respiratory failure secondary to COVID-19 pneumonia, incidentally, found to have marked leukocytosis. He was eventually diagnosed with CML. With this case report, we hope to educate clinicians on the approach for evaluating significant leukocytosis, diagnosis of a suspected hematologic disorder, and management of a newly diagnosed CML with concomitant COVID-19 infection.

## Case presentation

This is a 61-year-old male with no significant past medical history who presented for evaluation of hypoxia. The patient’s home pulse oximeter revealed an oxygen saturation ranging in the mid to high 80% for the past day prior to admission. He also complained of cough, worsening shortness of breath, rhinorrhea, nausea, and vomiting of non-bloody and non-bilious contents for two weeks prior to admission. Of note, the patient tested positive for COVID-19, three days prior to admission. The patient was not vaccinated against COVID-19. 

Physical exam was notable for hypoxia on room air (oxygen saturation 88%), bilateral diffuse rales across all lung fields, and hepatosplenomegaly. The repeat COVID-19 test by polymerase chain reaction was positive. His laboratory results revealed marked leukocytosis (125 x 10^3^/µL), microcytic anemia, thrombocytopenia, elevated bands, myelocytes, metamyelocytes, and promyelocytes as seen in Table 1.

**Table 1:** Laboratory values on admission.

	Laboratory values	Reference Values
D-Dimer	809 ng/mL	<500 ng/mL
White Blood Cells	125 x 10^3^/µL	4.8-10.8 x 10^3^/µL
Hemoglobin	11.4 g/dL	14-18 g/dL
Platelet Count	87 x 10^3^/µL	150-400 x 10^3^/µL
Lymphocytes, percent	2.1%	20%-40%
Monocytes, percent	2.1%	0%-12%
Eosinophils, percent	3%	0%-8%
Segmented neutrophils, percent	50.5%	45%-70%
Bands, percent	12.4%	0%-10%
Metamyelocytes	10.3%	0%
Myelocytes	18.6%	0%
Promyelocytes	2.1%	0%
Blasts	2.1%	0%
Lactate Dehydrogenase	678 U/L	140-271 U/L

**Figure 1:**
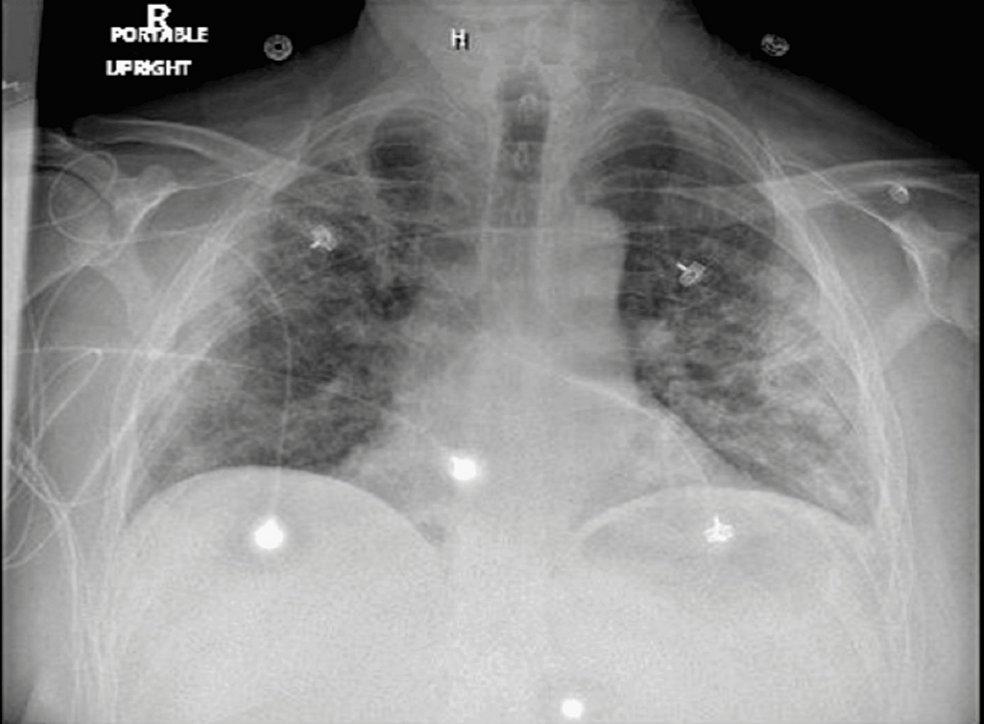
Chest x-ray showing patchy and confluent parenchymal infiltrates throughout the lungs.

The chest radiograph revealed diffuse patchy and confluent parenchymal infiltrates throughout the lungs (Figure 1).

Subsequently, leukemia/lymphoma peripheral blood flow cytometry revealed granulocytosis (92% of total cells) with partial aberrant CD56 expression and loss of CD13, CD16, and CD11b. Blasts were around 1% of total cells analyzed, expressing CD34, CD117, CD13, and human leukocyte antigen-DR (HLA-DR). B cells appeared polytypic, and T cells showed no aberrant loss of T-cell antigen, suggesting a myeloproliferative neoplasm. A subsequent fluorescence in-situ hybridization (FISH) showed a BCR-ABL1 fusion in 87% of cells confirming the diagnosis of CML.

The patient was placed on supplemental oxygen (5 liters nasal cannula), remdesivir for five days, and Intravenous dexamethasone 6 mg for 10 days. The patient was gradually weaned off supplemental oxygen and was able to maintain oxygen saturation in the mid to high 90% on room air on day 9 of hospitalization. The treatment for CML was deferred during his hospital stay. The patient was discharged after 12 days of hospitalization and was symptom-free. He was instructed to follow up with the local cancer center to establish care for the CML.

## Discussion

CML is a clonal hematopoietic stem cell neoplasm characterized by the overproduction of myeloid cells [5,6]. Secondary immunodeficiency with hematological malignancies has been well described in the literature [7]. The first systematic review evaluating COVID-19 hospitalization outcomes in immunocompromised patients concluded that immunocompromised patients have more comorbidities and worse outcomes compared to the general population [4]. However, specifically for CML, newly published literature suggests that patients with CML who become infected with severe acute respiratory syndrome coronavirus 2 (SARS-CoV-2) have lower mortality compared to other hematologic malignancies [8]. Our patient’s clinical course mirrors the new data found in the literature.

Most patients with CML present in the indolent or chronic phase [3,5]. Common symptoms, when present, are manifestations of anemia and splenomegaly. Splenomegaly is the most common physical finding, however, hepatomegaly, lymphadenopathy, and skin or subcutaneous lesions can also be observed [5]. The diagnosis of CML is first suspected by identifying the typical findings in the blood and bone marrow. CML diagnosis requires the demonstration of the Philadelphia chromosome, the BCR-ABL1 fusion gene, or the BCR-ABL1 fusion messenger ribonucleic acid (mRNA) by conventional cytogenetics, FISH analysis, or reverse transcription-polymerase chain reaction (RT-PCR) [3,9]. Our patient’s marked leukocytosis warranted further evaluation with peripheral blood flow cytometry, and subsequent FISH analysis to confirm BCR-ABL fusion. We suggest clinicians thoroughly work up any marked leukocytosis even when a leukemoid reaction is suspected due to severe infection.

Tyrosine kinase inhibitors (TKIs) are the initial treatment of choice for most patients with CML [10-12]. Hydroxyurea can be used to reduce white blood cell (WBC) counts while awaiting confirmation of a suspected diagnosis of CML in a patient with significant leukocytosis [12]. Our patient was not initiated on TKIs during hospitalization and was advised, upon discharge, to follow up with the local cancer center to establish care. Despite marked leukocytosis, our patient did not have any severe systemic symptoms of CML or symptomatic splenomegaly. The use of hydroxyurea was then dispensable. To our knowledge, there are no current guidelines about the initiation of TKIs or hydroxyurea during active COVID-19 infection. Our patient was initiated on conventional COVID-19 treatment, including steroids, despite marked leukocytosis, and had a favorable outcome.

## Conclusions

CML is a clonal hematopoietic stem cell neoplasm with the potential to cause secondary immunodeficiency in affected patients. Recent studies and clinical observations have demonstrated a poor outcome for immunodeficient patients hospitalized with COVID-19 infection. The approach to managing CML with concomitant COVID-19 pneumonia is still under review. With this case report, we hope to assist clinicians in the workup of marked leukocytosis in the setting of COVID-19 pneumonia and aim to help clinicians in the management of patients admitted with COVID-19 pneumonia and concomitant CML.
